# RETIRING TO POVERTY: VETERANS’ PROTESTS FOR NONPAYMENT OF PENSIONS IN NIGERIA

**DOI:** 10.1093/geroni/igad104.1990

**Published:** 2023-12-21

**Authors:** Runcie Chidebe, Agha A Agha, Ejike Ugwu, Donnette Narine, Takashi Yamashita, Phyllis Cummins

**Affiliations:** Miami University, Oxford, Ohio, United States; University of Nigeria Nsukka, Nsukka, Enugu, Nigeria; University of Nigeria Nsukka, Nsukka, Enugu, Nigeria; University of Maryland Baltimore, Baltimore, Maryland, United States; University of Maryland, Baltimore County, Baltimore, Maryland, United States; Miami University, Oxford, Ohio, United States

## Abstract

The lack of satisfactory retirement plans coupled with poor retirees’ well-being has led to the introduction of different pension and social security policies in Nigeria. Of all the public servants impacted by the weak pension system, Nigerian veterans are the most vulnerable. Veterans spent their youthful years serving the nation and defending its territory; however, when they retire, they retire to poverty. Since 2015, Nigerian veterans have been staging protests and blocking access roads for non-payment of pensions, gratuities, and other allowances. We adopted a qualitative research approach and used content analysis to examine 45 news articles (2015-2022) related to veterans’ protests for non-payment of pensions in Nigeria. A semi-structured interview was conducted with ten veterans. Our findings show the protests were perceived as the last resort to ‘save their lives’ from poverty and ill health. Non-payment of pensions to veterans in Nigeria has a negative socio-psychological impact on the veterans, their families, and the country. Some of the protest placards read: “We have sacrificed our youthful years fighting for Nigeria, we should be celebrated’, “When we remember the dead, we should also remember the living”. Some quotes from the interviews are: “We’re here, alongside our wives and children, and the widows of late military personnel and veterans who died in service, some of whom died fighting Boko Haram terrorists. We’ll be sleeping over at this place until they…accede to our demands.” Veterans organize themselves in diverse groups. Our findings provide evidence to support improved pensions for veterans in Nigeria.

